# Adapting physiologically-based pharmacokinetic models for machine learning applications

**DOI:** 10.1038/s41598-023-42165-3

**Published:** 2023-09-11

**Authors:** Sohaib Habiballah, Brad Reisfeld

**Affiliations:** 1 Department of Chemical and Biological Engineering, Colorado State University, Fort Collins, CO 80523-1301 USA; 2 School of Public Health, Colorado State University, Fort Collins, CO 80523-1612 USA

**Keywords:** Pharmacokinetics, Computational science

## Abstract

Both machine learning and physiologically-based pharmacokinetic models are becoming essential components of the drug development process. Integrating the predictive capabilities of physiologically-based pharmacokinetic (PBPK) models within machine learning (ML) pipelines could offer significant benefits in improving the accuracy and scope of drug screening and evaluation procedures. Here, we describe the development and testing of a self-contained machine learning module capable of faithfully recapitulating summary pharmacokinetic (PK) parameters produced by a full PBPK model, given a set of input drug-specific and regimen-specific information. Because of its widespread use in characterizing the disposition of orally administered drugs, the PBPK model chosen to demonstrate the methodology was an open-source implementation of a state-of-the-art compartmental and transit model called OpenCAT. The model was tested for drug formulations spanning a large range of solubility and absorption characteristics, and was evaluated for concordance against predictions of OpenCAT and relevant experimental data. In general, the values predicted by the ML models were within 20% of those of the PBPK model across the range of drug and formulation properties. However, summary PK parameter predictions from both the ML model and full PBPK model were occasionally poor with respect to those derived from experiments, suggesting deficiencies in the underlying PBPK model.

## Introduction

Machine learning is increasingly used in many aspects of drug discovery and development^1–3^, with a common use being the virtual screening of chemical libraries. Many of the studies published in the literature have focused on advances in screening for bioactivity^4–7^, with many fewer centered on pharmacokinetics^8–11^. These latter studies have proven to be relatively effective across large chemical libraries, but do not generally have the predictive power of special-purpose tools like physiologically based pharmacokinetic (PBPK) models.

PBPK models, which incorporates anatomical, physiological, and biochemical relationships, are widely used in the drug development process and have proven useful in predicting drug ADME for a range of therapeutics^12,13^, including pediatric drugs^14^, biopharmaceutics^15^, generic drugs^16^, monoclonal antibodies^17^, and nanoparticles^18^. In the case of orally administered drugs, compartmental absorption and transit (CAT) PBPK models are often used. These models include many details of drug absorption, metabolism, and transport in the gastrointestinal tract, which is often divided into discrete segments, each of which may account for drug in various states, e.g., unreleased, undissolved, dissolved, and absorbed into the enterocytes. Implementations of such models include the ACAT (advanced compartmental and transient) model^19^, part of the proprietary software GastroPlus^®^, and the OpenCAT model^20,21^, developed de novo using the open source software GNU MCSim^22^.

Usage of PBPK models, like ACAT or OpenCAT, requires the solution of (often complex) systems of differential equations that may make implementation difficult in certain workflows, like those common in machine learning pipelines^23^. One approach to address this limitations is to capture the essential features of PBPK simulation predictions within a trained ML model. Such a model could take as input drug-specific information and would output essential PK parameters as a stage in a multi-element analysis pipeline. The aim of this work was to explore the feasibility of this approach using OpenCAT as the underlying PBPK model.

## Methods

**Figure 1:**
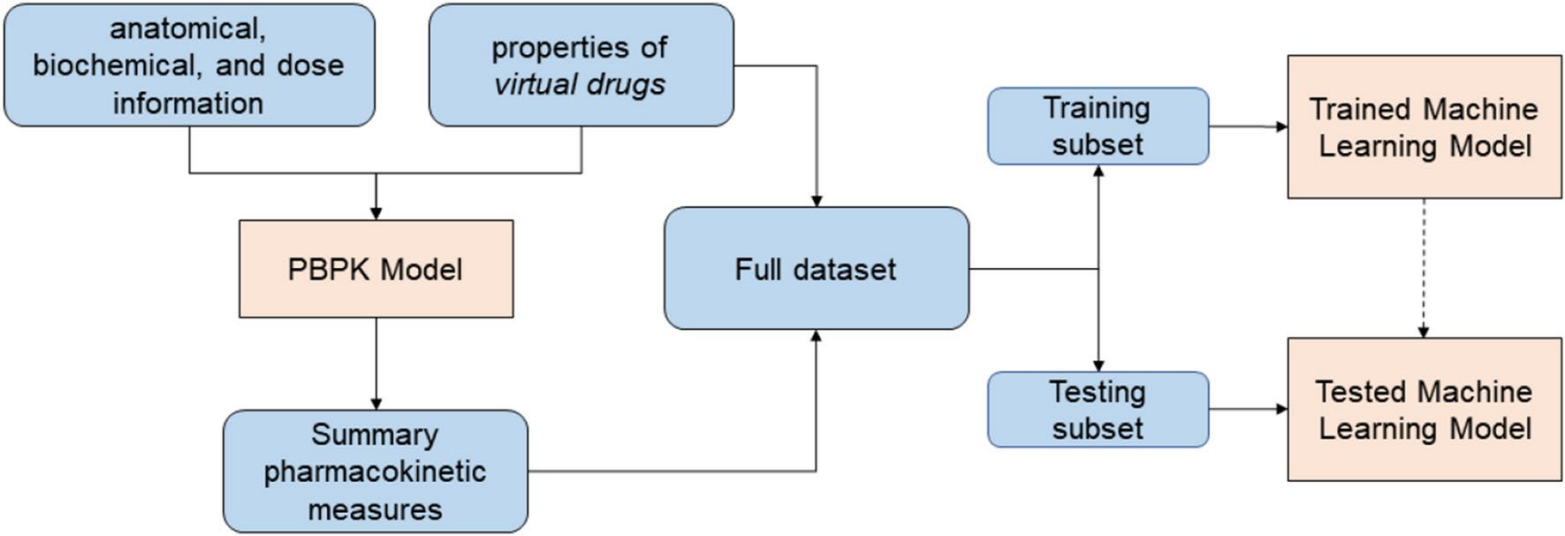
Elements and steps in the workflow.

To achieve the project aim, a machine-learning model was developed to take as input drug-specific properties and produce as output various summary pharmacokinetic measures comparable to those generated by the full PBPK model. The development workflow comprised several steps: (i) Generating a dataset comprising properties of a large set of *virtual drugs*, (ii) Simulating the pharmacokinetics of each member of the dataset using the PBPK model and calculating summary pharmacokinetic measures, (iii) Developing and training machine learning models using the drug properties and summary PK measures, (iv) Critically evaluating the models in terms of their predictive capabilities. A schematic of the workflow is shown in Fig. 1.

### Dataset generation

#### Drug properties

The aim of this part of the workflow was to generate a large set of *virtual drugs* whose properties spanned the four classes of the Biopharmaceutics Classification System (BCS)^24^, which organizes compounds into four categories: Class I—high permeability, high solubility; Class II—high permeability, low solubility; Class III—low permeability, high solubility; and Class IV—low permeability, low solubility. To generate these property sets, drug properties (molecular mass, molar volume, acidic dissociation constant, effective permeability, precipitation rate constant, drug solubility, particle radius, and drug density) and additional parameters influencing drug pharmacokinetics (the ratio of the drug unbound fraction over its partition coefficient ($FuPC_{\textrm{i}}$), metabolic parameters, dose magnitude, and subject body weight) were taken from the literature^25–31^ to establish realistic ranges for all measures (see Table 1). Assuming a uniform distribution for each of the ranges, a Monte Carlo sampling procedure was employed in which a given sample was constructed by drawing from each parameter distribution. In total, 15,000 *virtual drugs* were generated for use in subsequent steps in the workflow.

To assess whether the generated *virtual drugs* were representative of actual drugs, 40 drugs across all BCS classes were selected from ChEMBL^32^ and PubChem^33^ and their properties determined. Tests confirmed that 90% of the real drugs had at least one ’close match’ in the *virtual drugs* dataset and all of them had at least one ’moderate match’. In this analysis, ’close match’ and ’moderate match’ meant that all of the following properties of the *virtual drug* were within 15% and 50%, respectively, of those of an actual drug: molecular weight, density, molar volume, pKa, solubility, and effective permeability.

Though assigning drugs to a specific BCS classes is not always straightforward^34,35^, for the purpose of this work, classes were assigned based simply on specific thresholds of solubility and permeability^24,36^ Using this procedure, the library of virtual drugs comprised 6392 Class I, 2097 Class II, 4920 Class III, and 1591 Class IV drugs.

**Table 1:** Ranges for property of interest determined by examining physical properties of actual drugs spanning all BCS classes.

Parameter name	Range	Units
Molecular mass	20–1700	$\frac{{\text{g}}}{{{\text{gmol}}}}$
Molar volume	30–1200	$\frac{{\text{g}}}{{{\text{gmol}}}}$
Acidic dissociation constant	0–14	–
Particle radius	5–50	${\upmu {\text{m}}}$
Drug density	0.1–0.7	$\frac{{\text{g}}}{{{\text{ml}}}}$
Drug solubility	0.001–1000	$\frac{{{\text{mg}}}}{{{\text{ml}}}}$
Precipitation rate constant	0.01–1	h^-1^
Effective permeability of GI tract epithelia	0.015–8	$\frac{{{\text{cm}}}}{{\text{s}}}$
Unbound fraction over partition coefficient in compartment i, FuPC_i_	0.01–100	–
Subject’s body mass	45–125	kg
Dose magnitude	1–50 $\times$ 10^3^	$\upmu \text{mol}$
Metabolism rate constant, $K_{m}$	0.04–10	mM
Metabolism rate constant, $V_{max}$	10^-6^–10^-2^	$\frac{\upmu \text{mol}}{(\text{min}) (\text{mg}) (microsomal \;proteins)}$

#### Pharmacokinetic information

**Figure 2:**
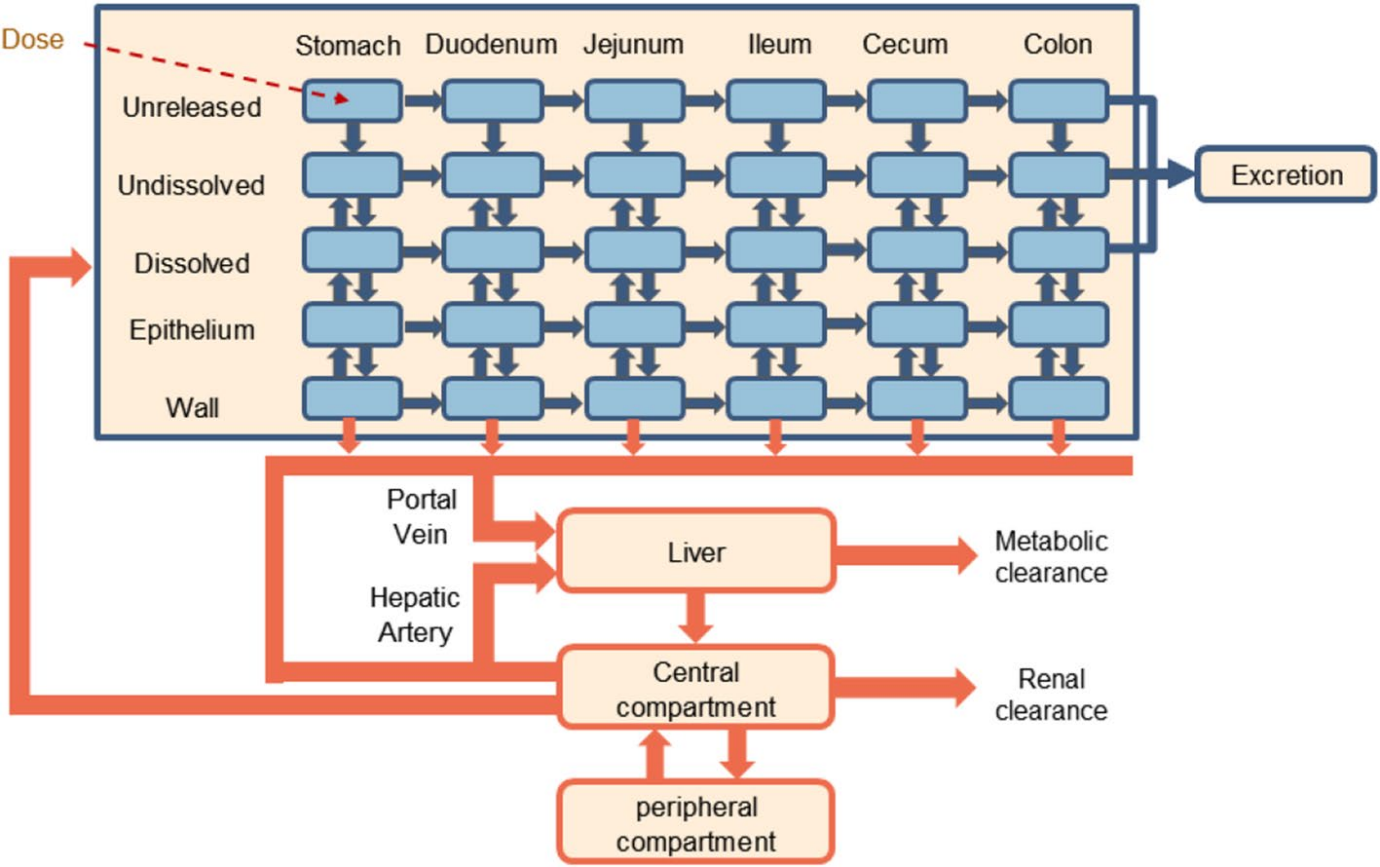
Structural overview of the OpenCAT PBPK model.

For each member of the set of *virtual drugs*, a simulation was performed using the OpenCAT model, whose structure is shown in Fig. 2. These simulations produced predicted time-course concentrations of the chemical in each state (unreleased, undissolved, dissolved) and compartment. For the purpose of comparison to experimental values from the literature, the detailed simulation results were condensed into the summary pharmacokinetic metrics (SPKMs) $C_{\textrm{max}}/dose$, $t_{\textrm{max}}$, and $AUC/dose$ using the predicted PK information from the blood. SPKMs values were normalized using min-max scaling.

Finally, to create the full dataset, the properties of each virtual drug were combined with its corresponding normalized SPKMs.

### Model development

Prior to model development, values were randomly selected from the full dataset (inputs + normalized SPKMs) to create training and testing subsets comprising 80% and 20% of the values, respectively. For each machine learning model, the drug properties and additional parameters noted earlier were used as *features*, while the normalized SPKMs were used as *labels*. Though a multivariate model could be constructed to predict all three normalized SPKMs, it was found that distinct models for each label (i.e., each normalized SPKM) consistently performed better in recapitulating target values.

Algorithm selection: To inform the process of algorithm selection, two machine learning algorithms appropriate for regression analyses, were evaluated: random forest^37^ and gradient boosting^38,39^. Preliminary evaluations indicated that both gradient boosting and random forest algorithm were equally suitable for this application, but because it proved to be more computationally efficient in cases of interest, random forest (RF) regression was selected.

Model training: Utilizing the training data set, the algorithm’s hyperparameters were tuned by performing a grid search. In addition to hyperparameters, the number of trees were optimized to maximize the accuracy of the algorithm while minimizing overfitting. From this process, the number of trees was set to 150 for all models.

### Model evaluation

Assessment metrics: To assess the performance of each ML-based model relative to those from the OpenCAT model, two metrics were used: (i) the relative error in predicted normalized SPKMs between the OpenCAT and ML models and (ii) the adjusted coefficient of determination across the entire set of *virtual drugs*, which is defined as

$$\begin{aligned} R^{2}\text {(adj)} = 1 - \frac{(1 - R^{2})(n - 1)}{n-k-1}, \end{aligned}$$

where *n* is the number of values in the data set, *k* is the number of independent features included, and $R^{2}$ is the the *unadjusted* coefficient of determination given by the conventional definition: $R^{2} = 1 - SSR / SST$, where *SSR* is the sum of squares of the residuals and *SST* is the total sum of squares.

Feature importance assessment: A study was conducted to evaluate the influence of feature set (number of features and features selected) on the accuracy of the model. Subsets having fewer features than the full feature set (FFS) are known as reduced feature sets (RFS). The RFS were generated iteratively and utilized the feature importance score^40^ for aggregation. The performance of each model was evaluated when trained using the FFS and all RFS. The *optimal* RFS was selected as the set with the highest $R^{2}\text {(adj)}$ value relative to the OpenCAT predictions.

Testing against experimental data: To further test the models, the normalized SPKMs were compared to those from OpenCAT and from experimentally-measured values for ten specific drugs (see Table 2).

**Table 2:** Drugs used for model evaluation.

Drug name	CAS number	BCS class	Literature references
Acetaminophen	103-90-2	Class III/IV	^41–43^
Codeine	76-57-3	Class I	^44,45^
Diazepam	439-14-5	Class I	^42,46^
Enalapril	75847-73-3	Class III	^47,48^
Fluvastatin	93957-54-1	Class II	^49,50^
Metoprolol	51384-51-1	Class I	^51,52^
Midazolam	59467-70-8	Class I	^53,54^
Ranitidine	66357-35-5	Class III	^42,55^
Trazodone	19794-93-5	Class II	^56,57^
Valacyclovir hydrochloride	124832-27-5	Class III	^58,59^

### Software

Python^60^ (v3.8) was used for general data processing. All machine learning simulations were conducted using scikit-learn (v1.1.3)^61^, and pharmacokinetic simulations were implemented and run using GNU MCSim^22^ (v6.1.0).

## Results

### Model verification

#### Full feature set model

**Figure 3:**
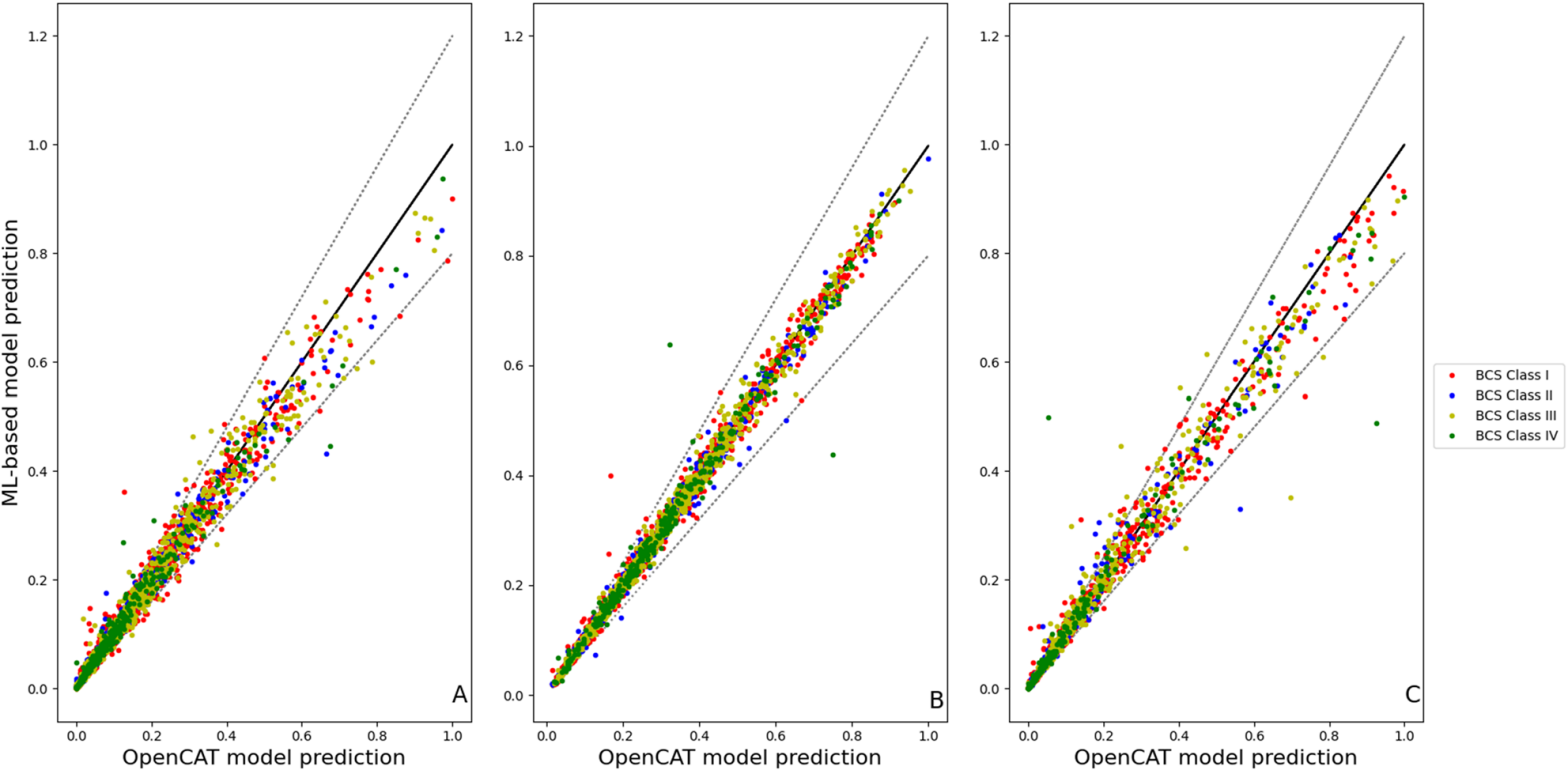
Comparison of normalized machine learning-based model predictions and their OpenCAT counterparts using the testing subset for (**A**) $C_{\textrm{max}}/dose$, (**B**) $t_{\textrm{max}}$, and (**C**) $AUC/dose$. In all panels, the solid line represents perfect agreement and the dashed lines indicate the ± 20% error bounds.

Figure 3 shows a comparison of the predictions of the full feature set (FFS) models with those from OpenCAT across the testing subset of *virtual drugs*. The $R^{2}\text {(adj)}$ values for the individual models were 0.85, 0.93, 0.86 for $C_{\textrm{max}}/dose$, $t_{\textrm{max}}$, and $AUC/dose$, respectively.

**Figure 4:**
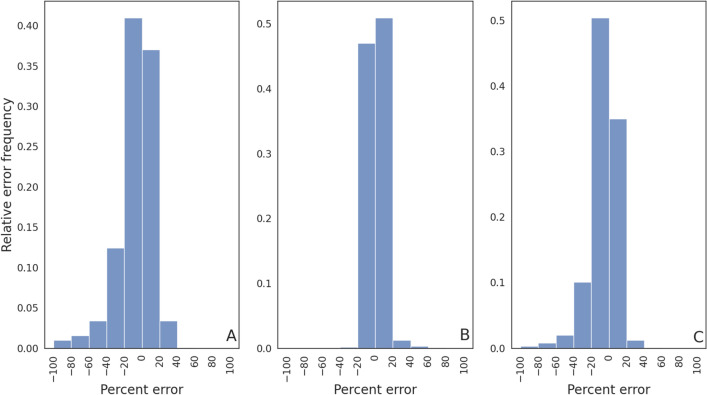
Relative error frequency for the full feature training set FFS for (**A**) $C_{\textrm{max}}/dose$, (**B**) $t_{\textrm{max}}$, and (**C**) $AUC/dose$.

To assess the degree of relative error between the machine learning and OpenCAT model predictions across the testing subset of *virtual drugs*, results were binned to create error frequency distributions (see Fig. 4). In the panels of this figure, the abscissa represents the relative error percentage, while the ordinate of the plots represents the relative error magnitude frequency. As summarized in Table 3, the total fraction of samples having relative errors in the range $\pm 20\%$ was 0.77, 0.97, and 0.82 for $C_{\textrm{max}}/dose$, $t_{\textrm{max}}$, and $AUC/dose$, respectively. At a threshold of $\pm 40\%$, the fractions were increased to 0.93, 0.99, and 0.95 for these same metrics.

#### Optimal reduced feature set model

Following the identification of the optimal reduce feature set, an evaluation identical to that for the FFS models was conducted. In this case, the $R^{2}\text {(adj)}$ values were 0.93, 0.98, and 0.95 for the $C_{\textrm{max}}/dose$, $t_{\textrm{max}}$, and $AUC/dose$ models, respectively.

**Figure 5:**
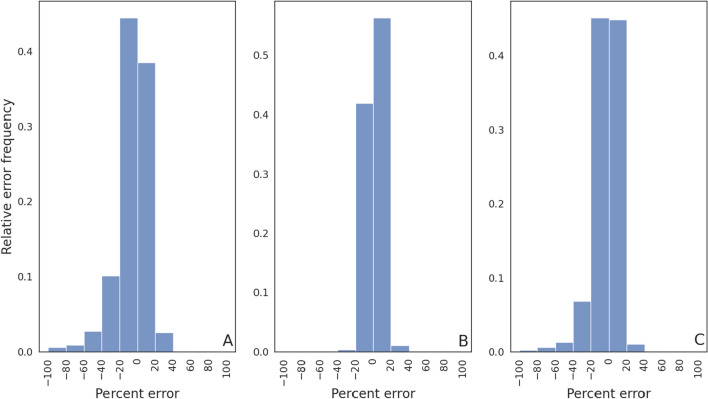
Relative error frequency for the optimal reduced feature training set (RFS) for (**A**) $C_{\textrm{max}}/dose$, (**B**) $t_{\textrm{max}}$, and (**C**) $AUC/dose$.

Similar to the previous case, histograms were generated to quantify the frequency of obtaining a prediction within a certain error percentage (see Fig. 5). As listed in Table 3, for this model the total fraction of samples having relative errors in the range $\pm 20\%$ was 0.83 for $C_{\textrm{max}}/dose$, 0.98 for $t_{\textrm{max}}$, and 0.90 for $AUC/dose$. For errors in the range of $\pm 40\%$, these fractions were increased to 0.96, 0.99, and 0.98.

**Table 3:** Fraction of predictions within relative error ranges for the full and reduced training set models.

Model	± 20%			± 40%		
	$C_{\textrm{max}}/dose$	$t_{\textrm{max}}$	$AUC/dose$	$C_{\textrm{max}}/dose$	$t_{\textrm{max}}$	$AUC/dose$
FFS	0.77	0.97	0.82	0.93	0.99	0.95
Optimal RFS	0.83	0.98	0.90	0.96	0.99	0.98

#### Feature importance

**Figure 6:**
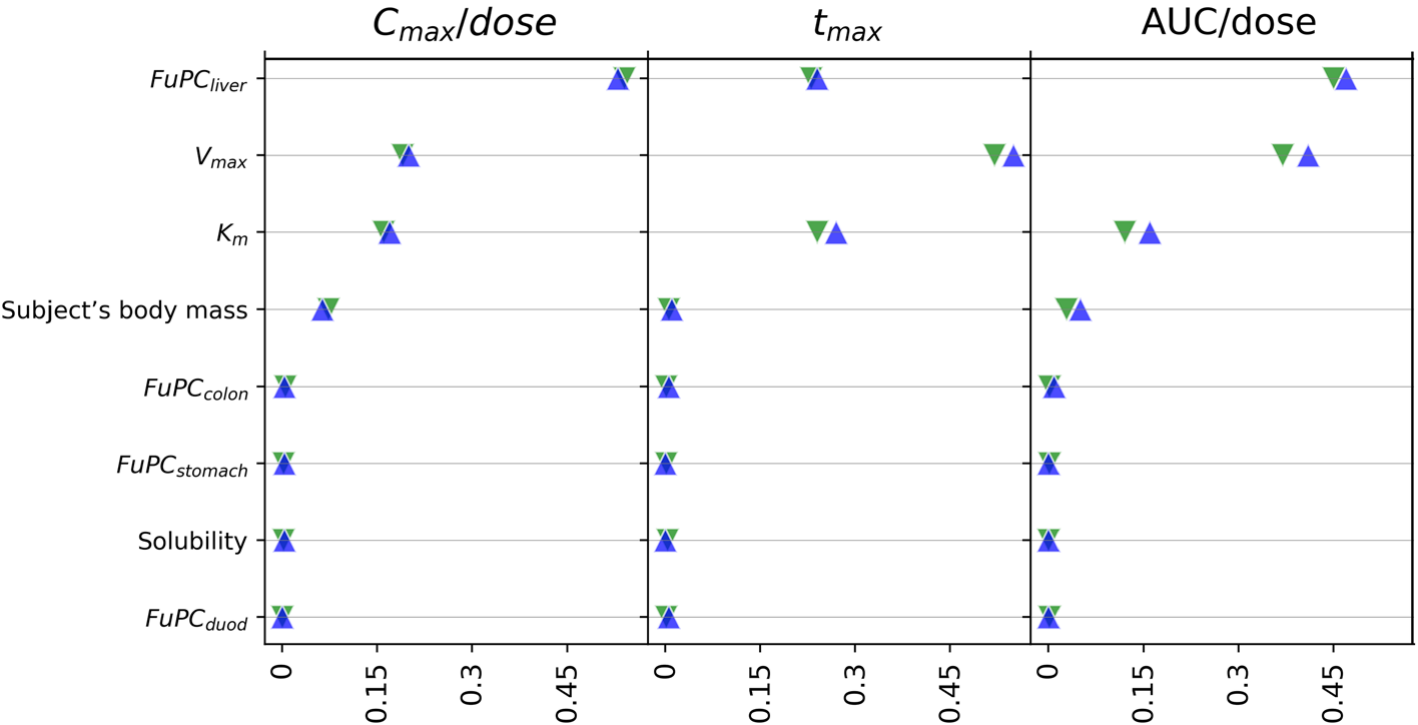
Feature importance assessment for the ML-based models. Green inverted triangles represent the feature importance scores associated with the FFS model while blue triangles represent the scores for the optimal RFS model.

As noted earlier, to assess the influence of each of the features on the model predictions, a feature importance study was conducted. It was found that the eight most influential features overall—and those used to create the optimal RFS model—were (i) the fraction unbound to partition coefficient ratio for the liver (FuPC_liver_), (ii, iii) the two metabolism rate constants ($V_{max}$ and $K_{M}$), (iv) the subject’s body mass, (v) the drug solubility, and (vi, vii, viii) the fraction unbound to partition coefficient ratio for the colon , stomach , and duodenum (FuPC_colon_, FuPC_stomach_, FuPC_duod_). Other results of this study are shown in Fig. 6, which depicts the feature importance scores for both the FFS and optimal RFS models.

### Model verification against experimental data

**Figure 7:**
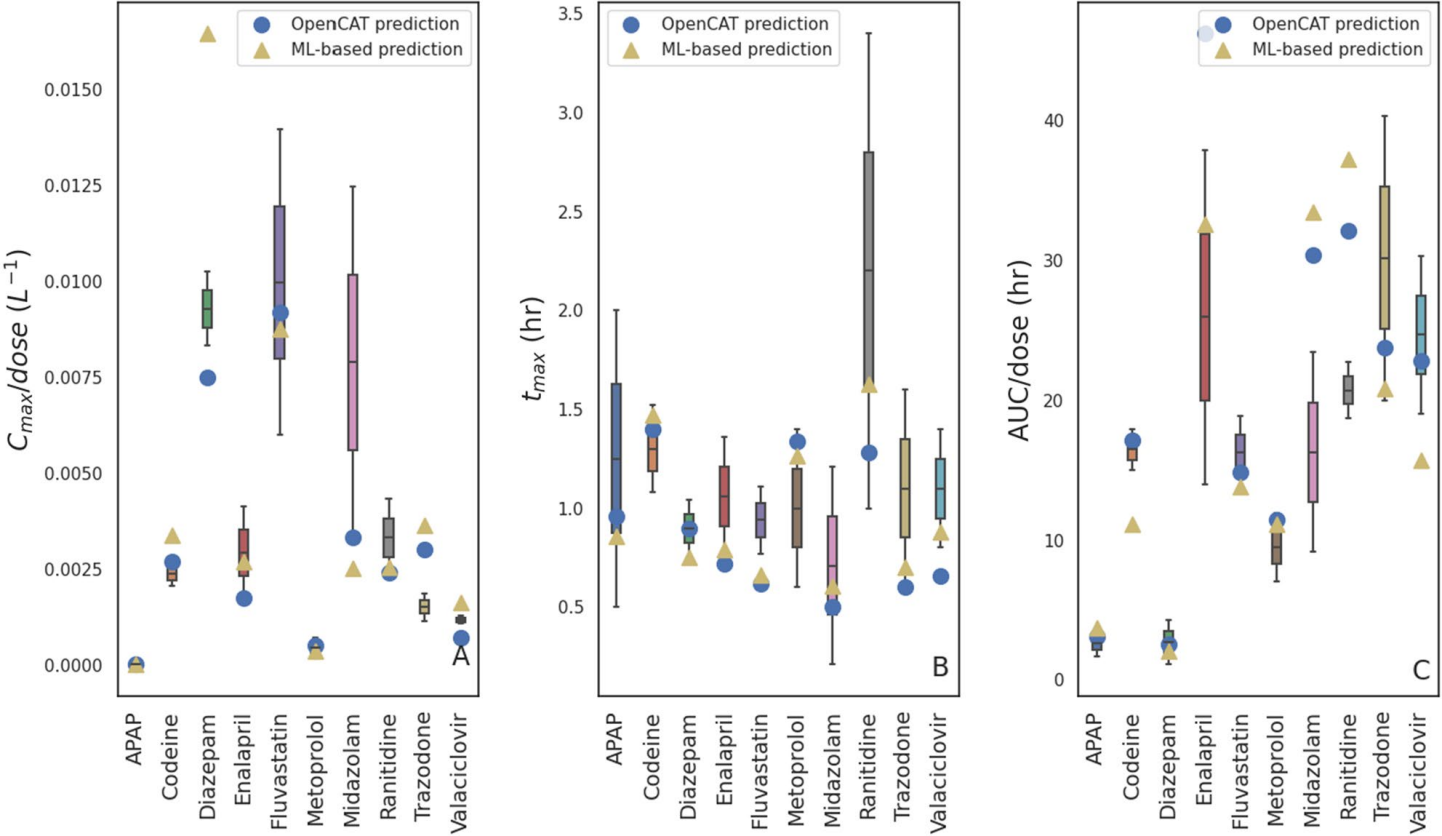
Comparison of experimental results vs those of the presents ML-based models and OpenCAT for (**A**) $C_{\textrm{max}}/dose$, (**B**) $t_{\textrm{max}}$, and (**C**) $AUC/dose$.

As an additional test, the optimized RFS models underwent comparison to the experimental data described earlier. The resulting values for the min-max normalized PK parameters are depicted in Fig. 7. There were often several values of the same SPKM from different experiments and/or cited uncertainty in these values. These values are represented in the figure as boxes (first to the third quartiles) and whiskers (minimum and maximum values). The predicted SPKMs from the ML and OpenCAT models are indicated by symbols. As expected, the agreement between the ML models and experimentally-obtained data was similar to that of the full PBPK model. While good agreement with experimental values was seen for many drugs and BCS classes ($R^{2}\text {(adj)}$ of 0.61, 0.79, and 0.77 for $C_{\textrm{max}}/dose$, $t_{\textrm{max}}$, and $AUC/dose$, respectively), models showed relatively poor predictive capabilities for others. These deficiencies may correspond to shortcomings previously described in the literature for the ACAT model for certain kinds of drugs^62^.

## Discussion

Agreement between predicted SPKMs for the machine learning models and full OpenCAT PBPK model were generally very good, suggesting that the methodology can be a viable means to introduce PBPK-level accuracy in pharmacokinetic predictions to a machine learning workflow. Models based on the optimized reduced feature set outperformed those based on the full feature set for all SPKMs evaluated, indicating that a feature optimization step is warranted to produce a model with the best fidelity with respect to the original PBPK model.

Though the primary focus here was on translating the OpenCAT model to a self-contained module appropriate as a component in a machine learning pipeline, it is expected that the methodology will be amenable to almost any PBPK model. Moreover, while this study focused on a specific set of model inputs and outputs (SPKMs), these can easily be customized for the application of interest.

Despite its promise, there are two potential deficiencies that must be considered. First, the generation of the set of *virtual drugs* used to underpin the methodology relied on randomly sampling values across parameter ranges. Although realistic values were used to establish these ranges, some combinations of properties likely resulted in unrealistic drug candidates. Second, the derived ML model will suffer the same predictive deficiencies and anomalies as the underlying PBPK model, so a prudent choice of the underlying model must be made.

## Data Availability

The datasets generated and/or analysed during the current study are available in a Zenodo repository, https://doi.org/10.5281/zenodo.7837360.
